# A Behavioral Change Smartphone App and Program (ToDo-CR) to Decrease Sedentary Behavior in Cardiac Rehabilitation Participants: Prospective Feasibility Cohort Study

**DOI:** 10.2196/17359

**Published:** 2020-11-03

**Authors:** Nicole Freene, Sander van Berlo, Margaret McManus, Tarryn Mair, Rachel Davey

**Affiliations:** 1 Physiotherapy University of Canberra Bruce Australia; 2 Health Research Institute University of Canberra Bruce Australia; 3 Onmi BV Eindhoven Netherlands; 4 Cardiology Canberra Health Services Garran Australia; 5 Exercise Physiology Canberra Health Services Garran Australia

**Keywords:** mHealth, eHealth, sedentary behavior, cardiac rehabilitation, mobile phone

## Abstract

**Background:**

Cardiac rehabilitation participants are encouraged to meet physical activity guidelines to reduce the risk of repeat cardiac events. However, previous studies have found that physical activity levels are low and sedentary behavior is high, both during and after cardiac rehabilitation. There is potential for smartphone apps to be effective in reducing sedentary behavior, although among the few studies that have investigated smartphone apps in cardiac rehabilitation, none targeted sedentary behavior.

**Objective:**

This study aims to evaluate the feasibility of a behavioral smartphone app (Vire) and a web-based behavior change program (ToDo-CR) to decrease sedentary behavior in cardiac rehabilitation participants.

**Methods:**

Using a single-center, pre-post design, participants were recruited by nursing staff on admission to cardiac rehabilitation. All eligible participants installed the Vire app, were given a Fitbit Flex, and received the 6-week ToDo-CR program while attending cardiac rehabilitation. The ToDo-CR program uses personalized analytics to interpret important behavioral aspects (physical activity, variety, and social opportunity) and real-time information for generating and suggesting context-specific actionable microbehavioral alternatives (Do’s). Do’s were delivered via the app, with participants receiving 14 to 19 Do’s during the 6-week intervention period. Outcome measures were collected at 0, 6, and 16 weeks. The assessors were not blinded. Feasibility outcomes included recruitment and follow-up rates, resource requirements, app usability (Unified Theory of Acceptance and Use of Technology 2 [UTAUT2] questionnaire), and objectively measured daily minutes of sedentary behavior (ActiGraph) for sample size estimation. Secondary outcomes included functional aerobic capacity (6-min walk test), quality of life (MacNew Heart Disease Health-Related Quality of Life Questionnaire), anxiety and depression (Hospital Anxiety and Depression Scale questionnaire), BMI, waist circumference, waist-to-hip ratio, and blood pressure.

**Results:**

Between January and May 2019, 20 participants were recruited consecutively. One-third of people who commenced cardiac rehabilitation were eligible to participate. Other than declining to take part in the study (15/40, 38%), not having a smartphone was a major reason for exclusion (11/40, 28%). Those excluded without a smartphone were significantly older than participants with a smartphone (mean difference 20 [SD 5] years; *P*<.001). Participants were, on average, aged 54 (SD 13) years, mostly male (17/20, 85%), and working (12/20, 67%). At 6 weeks, 95% (19/20) of participants were assessed, and 60% (12/20) of participants were assessed at 16 weeks. Participants were relatively satisfied with the usability of the app (UTAUT2 questionnaire). Overall, participants spent 11 to 12 hours per day sitting. There was a medium effect size (Cohen *d*=0.54) for the reduction in sedentary behavior (minutes per day) over 16 weeks.

**Conclusions:**

The use of a behavioral smartphone app to decrease sitting time appears to be feasible in cardiac rehabilitation. A larger randomized controlled trial is warranted to determine the effectiveness of the app.

## Introduction

In 2017, ischemic heart disease resulted in 8.93 million deaths worldwide and was the leading cause of years of life lost, which is a measure of premature death [1]. Physical inactivity and sedentary behavior are independent risk factors for cardiovascular disease, including ischemic heart disease, and all-cause mortality in both healthy and cardiovascular disease populations [2,3]. People with cardiovascular disease who watched television for 4 or more hours per day, a marker of sedentary behavior, were found to have a 52% increase in all-cause mortality compared with those who watched television for less than 2 hours per day [4]. Cardiac rehabilitation, a secondary prevention program, aims to reduce the risk of repeat cardiac events by targeting modifiable risk factors for ischemic heart disease, such as physical inactivity, smoking, and diet [5-7]. Despite strong scientific evidence for exercise-based cardiac rehabilitation decreasing morbidity and mortality in patients with heart disease, studies have found that physical activity levels are low in cardiac rehabilitation participants and sedentary behavior is high, approximately 8 to 11 hours a day [8-11].

The development of smartphone apps has been extensive in recent years, with many targeting healthy behaviors, including encouraging physical activity, offering health and exercise monitoring, motivation, and education [12,13]. At present, there is limited evidence regarding the effectiveness of smartphone apps in improving physical activity and sedentary behavior in healthy and heart disease populations [14-17]. The available evidence suggests that there is some potential for smartphone apps to be effective in increasing physical activity and decreasing sedentary behavior, with small effect sizes found [15,16]. This area is currently receiving increased attention in cardiovascular disease research [12,18].

In Australia, the country where this study was conducted, from 2016 to 2017, 91% of households accessed the internet via smartphones [19]. To investigate the use of mobile technology in cardiac rehabilitation, 282 Australian cardiac rehabilitation participants were surveyed from 9 hospitals and community sites in metropolitan and rural settings [18]. Approximately 65% of the people attending cardiac rehabilitation reported having a smartphone, with those aged <56 years being the biggest users of mobile apps (70%). Internationally, in Ireland and Belgium, 310 cardiac rehabilitation participants (mean age 62 years) were surveyed, and it was found that 97% of the patients had a mobile phone and 91% used the internet [20]. A total of 68% of the patients were interested in receiving cardiac rehabilitation support via a mobile phone. Despite the high use of mobile phones in cardiac rehabilitation, few studies have investigated the efficacy of smartphone apps, excluding text messaging–only interventions, in this population [16].

One such study compared cardiac rehabilitation delivered via a smartphone app with traditional center-based cardiac rehabilitation and addressed a number of risk factors [21]. They found that there is potential for cardiac rehabilitation to be delivered via a smartphone app (intervention group) as an alternative to traditional programs. However, despite reporting that 89% of the intervention group (smartphone app group) recorded daily physical activity, they failed to directly report on physical activity levels in either group or whether this had changed over time. In contrast, another study investigated a personal health assistant delivered via the web and smartphone-based platforms in addition to cardiac rehabilitation and encouraged the adoption of healthy lifestyle behaviors, including physical activity [22]. Cardiac rehabilitation participants at the beginning of cardiac rehabilitation and after 3 months of attending cardiac rehabilitation were divided into intervention and control groups (nonrandomized, 4 groups). Compared with the control groups, the personal health assistant group had significantly decreased weight (*P*=.03) and blood pressure (*P*=.01), with no difference in self-reported physical activity (*P*=.24). Notably, both intervention groups showed significant reductions in rehospitalizations and emergency department visits during the study period compared with the cardiac rehabilitation–only groups (*P*<.05). Another study, using a randomized multicenter design, evaluated a smartphone-based interactive tool for heart attack participants attending secondary prevention programs to assess whether it had an impact on lifestyle changes, including physical activity, and drug adherence [23]. The app included personalized feedback messages, using a traffic light model to describe the participant’s status on whether or not they were adhering to the medical recommendations, according to the data they entered. At 6 months, there was greater drug adherence in the app group, but there was no difference in self-reported lifestyle modifications, including physical activity.

With high levels of sedentary behavior reported in cardiac rehabilitation participants and low levels of physical activity, new initiatives are needed to improve the effectiveness of cardiac rehabilitation programs to address these behaviors. In addition, interventions aiming to decrease sedentary behavior appear to be more effective if they focus on sedentary behavior and not physical activity or a combination of both, and this should be taken into consideration [24]. There is some evidence that smartphone apps are able to modify risk factors for heart disease in cardiac rehabilitation populations [21-23] and interventions using computer, mobile, and wearable technologies can be effective in reducing sedentary behavior in healthy populations, but the evidence is limited [25]. No studies have investigated the use of a smartphone app to reduce sedentary behavior in cardiac rehabilitation participants. Therefore, the main aim of this study is to conduct a feasibility study as a precursor for a larger randomized controlled study to determine whether the behavioral smartphone app (Vire) and web-based behavior change program (ToDo-CR) targeting sedentary behavior are feasible in cardiac rehabilitation participants [26]. Specifically, the aims are as follows:

1. To evaluate the feasibility of the smartphone app (Vire) and web-based behavior change program (ToDo-CR) in cardiac rehabilitation, including recruitment, response and follow-up rates, and the usability of the app.
2. To estimate the sample size for a larger randomized controlled trial based on the SD of the main outcome measure (sedentary behavior) [27].

## Methods

### Design

This feasibility study was a single-center, pre-post design study conducted over 16 weeks at the Canberra Hospital (Australia) cardiac rehabilitation program (Australian New Zealand Clinical Trials Registry: ACTRN 12617001429347). Participants were assessed on admission to cardiac rehabilitation, at the end of the 6 week program, and at 16 weeks after admission to the program. The phase 2 cardiac rehabilitation program is multidisciplinary, time limited (12 sessions; 2 per week for 6 weeks), conducted in groups, hospital based, and has educational and supervised exercise components (1 hour education and 1 hour exercise). Ethics approval was received on February 14, 2018, from the Australian Capital Territory Health Human Research Ethics Committee (ETH.10.17.230). Study information, including the project aim; data storage; and details regarding participant involvement, confidentiality, and anonymity, were provided to participants at the beginning of the study. All participants provided written informed consent after reading this information.

### Recruitment

Cardiac rehabilitation staff recruited consecutive participants who commenced cardiac rehabilitation between January and May 2019. Eligible participants were those aged ≥18 years, currently enrolled in the cardiac rehabilitation program, and who had a smartphone. Participants were included if they had stable coronary heart disease (CHD) and were receiving optimal medical treatment with or without a revascularization procedure, that is, coronary artery bypass graft surgery, percutaneous coronary intervention (PCI), or myocardial infarction. Participants were excluded if they had a primary diagnosis of atrial fibrillation, New York Heart Association class II-IV symptoms of heart failure, uncontrolled arrhythmias, severe chronic obstructive pulmonary disease, uncontrolled hypertension, symptomatic peripheral artery disease, unstable angina, or uncontrolled diabetes; if they were unable to perform a submaximal walking test or unable to wear an accelerometer because of disability, for example, if they were confined to a wheelchair; and if they did not have adequate English language and cognitive skills. Participants were also excluded if they had a prepaid phone plan (limited data availability) or if the smartphone’s operating system was not compatible with all apps.

### Intervention

On the first day of attendance at the cardiac rehabilitation program, eligible participants were given a wrist-worn Fitbit Flex that could be worn for 24 hours and written information on how to install the Vire and Fitbit apps on their smartphones. To access the Vire app, participants needed to use a study-specific log-in code. Participants were requested to wear the Fitbit Flex for the 16-week study period and were able to keep the Fitbit Flex on completion of the study.

ToDo is a cloud-based behavior change program delivered through a smartphone app (Vire) created by Onmi in collaboration with Do Something Different Limited [28,29]. The Vire app has been progressively developed over the course of several projects together with end users and health care professionals [28]. A previous version of Vire used a co-design method called Experiential Design Landscapes [30]. This version of the Vire app had similar features for listing, opening, and completing context-specific actionable microbehavioral alternatives (Do’s) and was further refined using the Klikker methodology [31]. The Klikker methodology aims to unite the designer, developers, and end users in the initial phases of development by using modern web technologies, readily available and interchangeable design, and analytics software. Klikker combines the collection of quantitative user behavior and qualitative feedback from end users on their own devices to support the design process for researchers and designers. The Vire app used in ToDo-CR is created through another iteration of design and development, keeping in mind some basic principles of persuasive design. This version is substantially simplified based on user feedback. It reduces cognitive load by reducing the amount of information presented at once. The app is more appealing, information is decluttered, and visual consistency and hierarchy have been improved. The navigation is slightly simplified by limiting options to 4 options and prioritizes the dynamic home screen in an attempt to conserve attention and engagement. The Vire app is available in both the iOS and Android versions.

**Figure 1:**
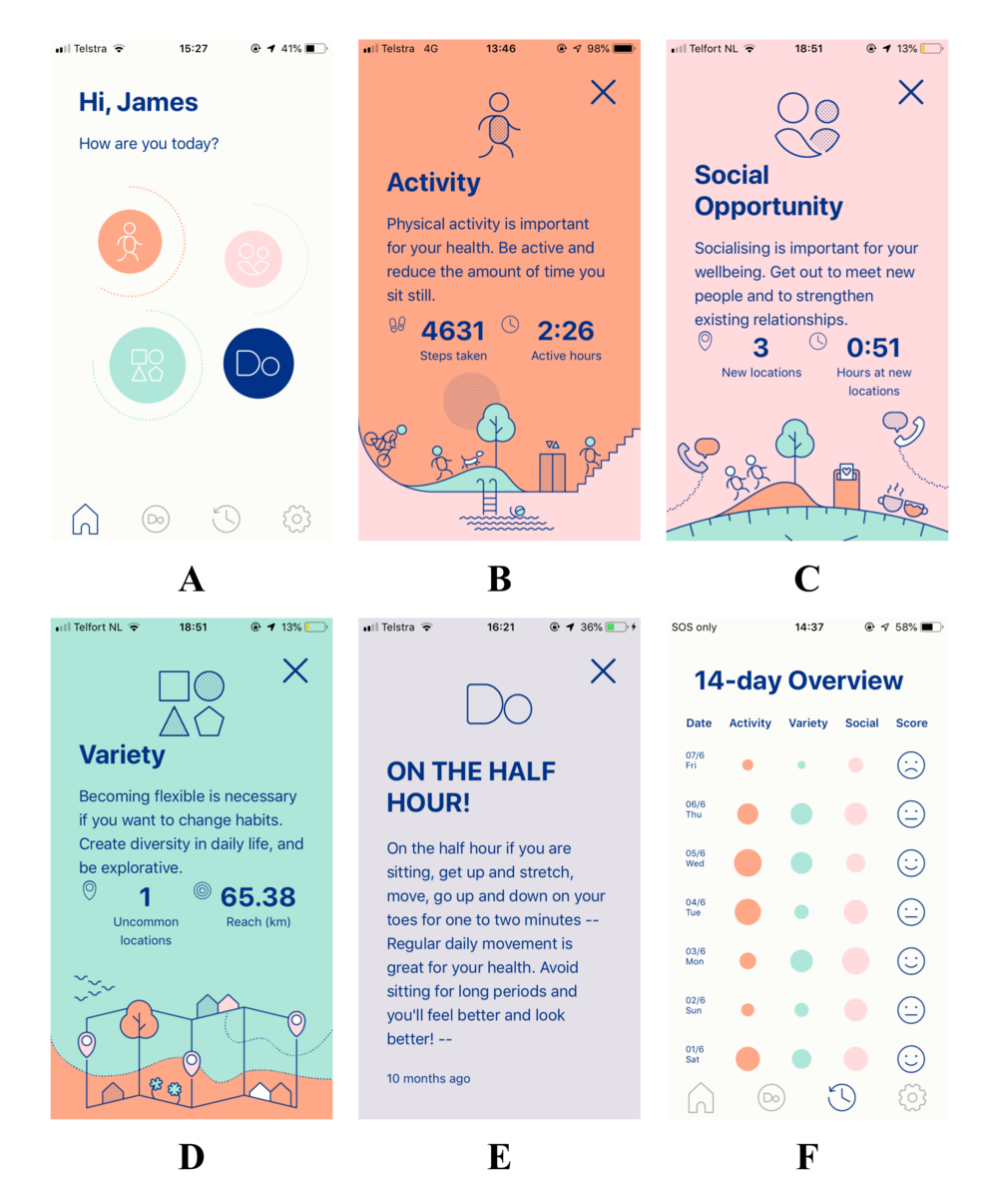
User interface of the app.

The ToDo program aims to improve an individual’s behavioral flexibility, learning new behaviors so they have more choice over how they react to different situations [32]. The program suggests microbehavioral alternatives (Do’s) that gradually change people’s habits, with some evidence that these small behavioral changes, which may not directly target the habit of interest, effect health outcomes such as decreases in weight [28,32]. The original program has been adapted by the research team to target sedentary behavior, based on Australian physical activity and cardiac rehabilitation guidelines to create ToDo-CR, a 6-week behavior change program (Figure 1) [5,33]. By combining technology, evidence-based guidelines, and behavior change techniques such as action planning and feedback [34], the ToDo-CR program aims to increase the participants’ self-efficacy and behavioral flexibility and decrease their sitting time.

The ToDo-CR program is personalized and consists of different types of Do’s delivered through the smartphone app via push notifications: Core Do’s and Data-Driven Do’s. The Do’s are small actionable and achievable goals based on the individual’s data. They provide prompts on how to achieve the goal and opportunities to practice new behaviors (Figure 1E). Core Do’s address the individual’s existing habits that often prevent healthy changes. Data are used from the answers to a questionnaire completed in the app at the start of the program asking questions about risk factors and desired behaviors, for example, “how often do you spend most evenings watching TV or in front of a screen?”, determining the Core Do’s that are distributed. Data-Driven Do’s address the individuals’ everyday context that traps them in habitual behavior by combining data from the Fitbit Flex (activity data) and the Vire app (GPS) to create a comprehensive digital profile of the individual. Real-time analysis algorithms use the GPS and activity data to calculate scores in 3 main variables: physical activity, social opportunity, and variety, and providing feedback to participants, allowing self-monitoring, and aiming to increase their self-efficacy (Figure 1A-D). Physical activity measures steps per day and the amount of time spent being active (Figure 1B). Social opportunity uses GPS coordinates to extract the number of new places visited and the amount of time spent in these places, and by combining these 2 parameters to estimate the chances of meeting people, the program encourages participants to change their social environment (Figure 1C). Variety uses GPS coordinates as well as activity data, including uncommon places visited, the distance traveled, the routes taken, and the order and time at which places are visited, thus analyzing how much the individual’s day differs from an average day and encouraging participants to change their physical environment (Figure 1D). For all variables, a baseline assessment is conducted for 1 week at the start of the program to understand a person’s routine and activity capabilities. The baseline computation includes an assessment of the minimum and maximum values recorded during the week. The parameters are then linearly rescaled in a 0 to 10 range using the information collected in the baseline assessment (minimum and maximum value). The *0* value is assigned to the daily values that are equal or below the minimum, and the value *10* is assigned to the daily values that are equal or exceed the maximum value registered during the baseline period. The scores for each variable are made relative to each participant, and the 0 to 10 range represents different levels of activity. Therefore, individuals are only prompted to make relative improvements, not to reach absolute levels. Scores for each variable are represented by the size of the circle on the home page (Figure 1A) and in the 14-day overview (Figure 1F). The larger the circle, the higher the score, indicating a greater change from the individual’s baseline measures.

The Data-Driven Do’s within the ToDo-CR program are dispatched based on these measurable variables or habits. Before sending any Data-Driven Do’s, the program checks intraday data to ensure that the analysis represents the day sufficiently, that is, data must represent more than 60% of the total available data to be considered precise enough to dispatch a Do. The system logs were continuously monitored using automated methods and manually for errors. When the participants’ scores were low on 3 consecutive days, an individualized, context-specific Data-Driven Do was sent to stimulate the participant to improve their score and behavior, and it provided an opportunity for participants to mark the Do as completed (Figure 1E). Participants received feedback on their daily variable scores within the 14-day overview by receiving sad, neutral, or smiley faces to reward them for changes in their behavior and allowed them to track observed trends (Figure 1F). In total, there were 89 different Do’s, small actionable and achievable behavioral goals, which could be dispatched to individuals depending on their individualized data, a combination of Core Do’s and Data-Driven Do’s. One-third (30/89, 38%) of the Do’s targeted decreasing sedentary behavior and increasing physical activity. The maximum number of Do’s received by participants per week was 3, with participants receiving 14 to 19 individualized Do’s during the 6-week intervention period. Some of the Do’s contained hyperlinks to other resources, such as the Australian Heart Foundation website [35]. During the study period, 5 updates were performed to improve location tracking and the functionality of the app. The content of the Do’s and analysis of the behavioral variables did not change during the study period. Participants had access to the Vire app for the entire 16 weeks.

### Outcome Measures

All assessments were conducted at the hospital and were carried out by a cardiac rehabilitation nurse, exercise physiologist, or physiotherapist, who were not blinded. The main feasibility outcome measures were the number of eligible participants, follow-up rates and response rates to questionnaires, and the usability of the app (the Unified Theory of Acceptance and Use of Technology 2 [UTAUT2] questionnaire) [27]. Objectively measured sedentary behavior was used to estimate the sample size for a larger randomized controlled trial. Other outcome measures included objectively measured moderate-to-vigorous physical activity (MVPA), BMI, waist-to-hip ratio, blood pressure, exercise capacity (6-min walk test, 6MWT), quality of life (MacNew Heart Disease Health-Related Quality of Life Questionnaire, MacNew), anxiety and depression (Hospital Anxiety and Depression Scale, HADS), and clinical and demographic information.

#### Smartphone App Usability and Adherence

The UTAUT2 questionnaire was used to assess the usability of the Vire app and ToDo-CR program at 6 and 16 weeks [36]. The UTAUT2 was developed as a comprehensive integrated model for better understanding consumer acceptance of a new technology or system and has been used in adults with multiple chronic conditions [37]. UTAUT2 is a 23-item self-reporting questionnaire consisting of 7-point Likert-scale items. The items assess the following constructs: performance expectancy, effort expectancy, social influence, facilitating conditions, hedonic motivation, habit, and behavioral intention. Behavioral intention is expected to have a significant influence on the use of the smartphone app [38]. In addition, the completion of Do’s as marked by the participant was used as an indicator of adherence to the program.

#### Sedentary Behavior and Physical Activity

A triaxial commercial accelerometer (ActiGraph, ActiSleep) was used to objectively assess sedentary behavior and physical activity. Participants were asked to wear the monitor on their right hip during waking hours for 7 consecutive days and not to wear the accelerometer in water. All data were sampled and downloaded as raw data (30 Hz) and converted to 15-second epochs (time interval) and then to counts per minute (cpm) using the ActiLife software [9,10]. Data were screened, excluding data if less than 10 hours per day wear time (nonwear defined as >60 consecutive minutes where there is zero activity, with no allowance of epochs with counts above zero) and less than 4 days of valid data [9,10,39]. If there were more than 7 days of valid data, all valid days were used to calculate the average [10]. The Sasaki vector magnitude cutpoints were used to determine the time spent in light (150-2689 cpm) and MVPA (≥2690 cpm) [9,10,39,40]. To measure the sedentary behavior, the vector magnitude cutpoint was used (<150 cpm) [9,10,39,41]. Estimating daily time spent in physical activity and sedentary behavior was calculated by dividing the total time spent (in minutes) in each threshold by the number of valid days. In addition, daily time spent in sedentary behavior was expressed as a percentage of the total daily wear time. Sedentary behavior bout data used a minimum length of 10 min, with no drop time, recording the number of sedentary bouts per day [9]. The average sedentary bout length and number of sedentary breaks were also recorded.

#### Anthropometric Characteristics and Blood Pressure

Height (m), weight (kg), and BMI (kg/m^2^) were recorded using a calibrated set of scales and a stadiometer. Waist and hip circumference were measured in centimeters using a tape measure. Blood pressure levels were obtained using a standardized sphygmomanometer on the right arm of seated subjects.

#### Exercise Capacity

The 6MWT is a commonly used objective measure of functional exercise capacity in cardiac rehabilitation [42]. The distance an individual was able to walk along a flat 25 to 30 m walkway over a 6-min period was recorded. The test is a self-paced, submaximal test of exercise capacity and has been found to have moderate to high reliability and validity [42].

#### Health-Related Quality of Life

The MacNew was used for the assessment of heart disease–specific health-related quality of life. The MacNew is self-administered and consists of 27 items that fall into 3 domains (physical limitations, 13 items; emotional function, 14 items; and social function, 13 items). The maximum possible score in any domain is 7 (high health-related quality of life), and the minimum possible score in any domain is 1 (poor health-related quality of life). The time frame for the MacNew is the previous 2 weeks, and it has good reliability and validity internationally [43].

#### Anxiety and Depression

The HADS was used to assess anxiety and depression [44]. This questionnaire is a 14-item self-reporting questionnaire comprising 4-point Likert-scale items covering the occurrence of symptoms of anxiety and depression over the 2 weeks before taking the questionnaire. Each item on the questionnaire is scored from 0 to 3, so that a person can score between 0 (best outcome) and 21 (worst outcome) for either anxiety or depression. The HADS has demonstrated excellent discriminant validity, construct validity, test-retest reliability, and internal consistency in adults with cardiovascular disease [45].

#### Demographic and Clinical Questionnaire

Participants were assessed on their sociodemographic variables (ie, gender, age, education level, relationship status, and current employment status) and clinical predictor variables (ie, smoking status and other medical conditions).

### Statistical Analysis

As this is a feasibility study, a formal sample size calculation was not completed [27]. The aim was to recruit a minimum of 20 participants. All participants who completed the baseline assessment and attended at least one cardiac rehabilitation session were included in the sample. Intention-to-treat analysis was performed. For missing data at follow-up, the last value was brought forward. Descriptive analyses were completed. The normality of the data was assessed using the Kolmogorov-Smirnov test. For data that were normally distributed, repeated-measures analysis of variance was used to test for differences within the cohort. If variables were not normally distributed, the Friedman test was used. For accelerometer data, differences in wear time were controlled for by using individual mean wear time (within-subject effects). The significance level was set at *P*<.05. All data were analyzed using SPSS, version 25.

## Results

### Recruitment and Response Rates

**Figure 2:**
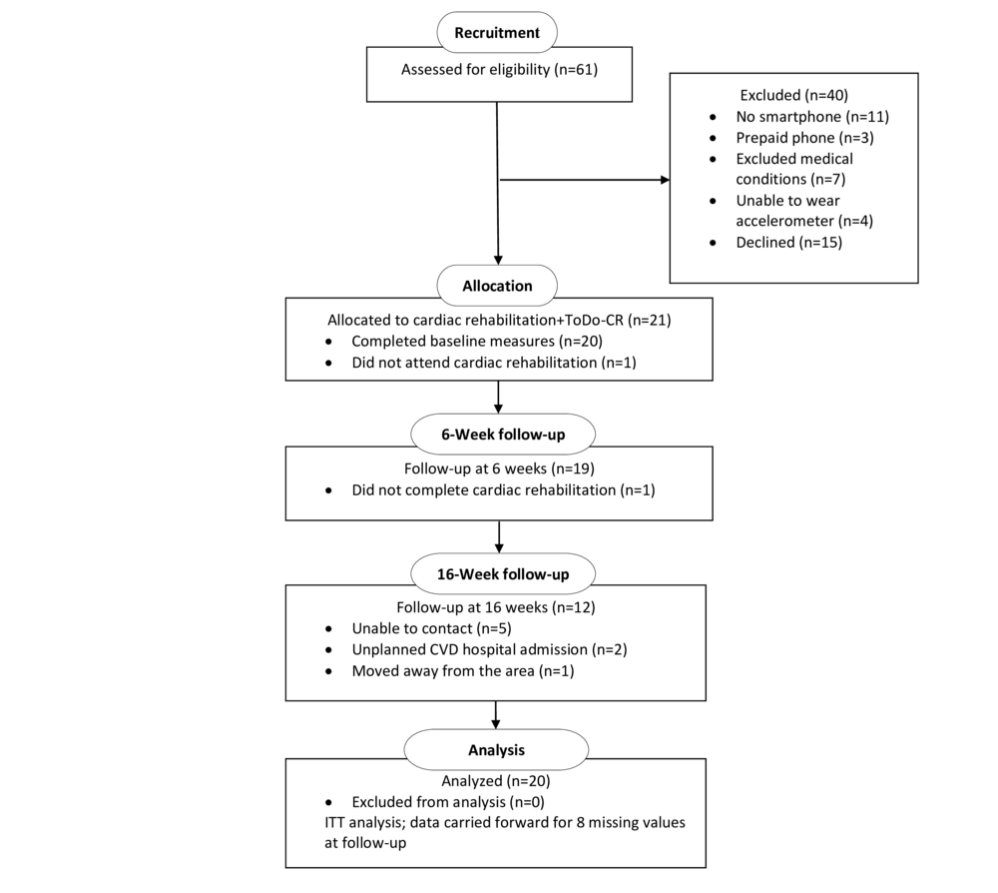
Flow of participants through the ToDo-CR feasibility study. CVD: cardiovascular disease; ITT: intention-to-treat.

A total of 20 participants were consecutively recruited for this feasibility study (Figure 2). One-third (21/61, 34%) of the people with CHD who commenced cardiac rehabilitation over the 4-month recruitment period were eligible to participate in this study. Other than declining to take part in the study (15/40, 38%), not having a smartphone was a major reason for exclusion (11/40, 28%). Those excluded without a smartphone were significantly older than participants with a smartphone (*P*<.001). Participants were, on average, aged 54 years, mostly male, in a relationship, and working (Table 1). Most participants had undergone a PCI, were nonsmokers, and did not have type 2 diabetes or other chronic diseases, and half of them were tertiary educated. A total of 85% (17/20) of participants attended all cardiac rehabilitation sessions during the 6-week cardiac rehabilitation program. At follow-up, 95% (19/20) of participants were assessed at 6 weeks, and 60% (12/20) of participants were assessed at 16 weeks (Figure 2). A quarter (5/20, 25%) of the participants were unable to be contacted at 16 weeks. Moreover, 2 participants had unplanned cardiovascular disease hospital admissions and were unable to complete their final assessment at 16 weeks.

**Table 1:** Characteristics of participants at baseline (N=20).

Characteristics		Values
Age, years, mean (SD)		54 (13)
Gender, male, n (%)		17 (85)
Country born, Australia, n (%)		11 (61)
Paid work, full or part time, n (%)		12 (67)
Education level, tertiary, n (%)		9 (53)
Relationship status, partner, n (%)		14 (78)
**Diagnosis, n (%)**		
	Coronary heart disease	2 (10)
	Percutaneous coronary intervention	16 (80)
	Myocardial infarction	1 (5)
	Coronary artery bypass graft	1 (5)
Other chronic disease (no), n (%)		12 (67)
Current smoker (no), n (%)		16 (89)
Type 2 diabetes (no), n (%)		15 (83)

### App Usability and Adherence

Participants were relatively satisfied with the usability of the app at 6 weeks, with median scores in all constructs greater than 4, except for *habit* (Table 2). However, satisfaction with the app declined at 16 weeks after the Do’s ceased at 6 weeks. There were significant positive correlations (*P*<.05) between all UTAUT2 constructs and *behavioral intention to use* the app except for *effort expectancy* at 6 weeks. This indicates that the ease of use of the app may not be a factor in the intention to use the smartphone app. At 16 weeks, only *performance expectancy* (*r*=0.70; *P*=.02) and *habit* (*r*=0.80; *P*=.003) had significant correlations with *behavioral intention*, indicating that participants believe that the smartphone app will help them to make changes in their behavior, and the use of the smartphone app has become a habit that influences their intention to use smartphone apps in the future. In terms of adherence, 73.7% (252/342) of the Do’s sent to participants during the 6-week intervention period were marked as completed.

**Table 2:** Smartphone app usability (the Unified Theory of Acceptance and Use of Technology 2 questionnaire constructs) at 6 and 16 weeks.

Construct (7-point Likert scale^a^)	6 weeks (n=15), median (IQR)	16 weeks (n=12), median (IQR)
Performance expectancy	4.25 (2.5-5.25)	1.75 (1-2.94)
Effort expectancy	4.5 (3.75-5.75)	3.5 (3-4.25)
Social influence	4 (3.5-4.17)	2 (1-4)
Facilitating conditions	4.88 (4.19-6.06)	4.75 (3.75-6.25)
Hedonistic motivation	4.17 (3.83-5)	1.5 (1-3.25)
Habit	3.75 (2-4.25)	1 (1-3)
Behavioral intention	4 (2.33-6)	1 (1-4.33)

^a^Likert scale: 1=strongly disagree; 4=neutral; and 7=strongly agree.

### Resource Requirements

All participants installed the app, were given a Fitbit, and received the 6-week ToDo-CR program. Participants required more support than expected to install the app, to link the app to the Fitbit app, and to troubleshoot any issues with the app and Fitbit. The Vire app required updating in the initial stages of the study, which caused some issues. Consequently, written material was developed to support this, and a frequently asked questions button was added to the app. The research assistant also called all participants within the first week of commencing the study to determine if they were having any issues with the app and provided advice and support accordingly. In addition, during recruitment, some nursing staff were unsure about introducing the app to potential participants and checking whether or not the smartphones of potential participants had suitable operating systems to be eligible for this study. Simplified written material and instructions on downloading the Fitbit app were developed to aid nursing staff and to ensure that the recruitment process was as efficient as possible to decrease the impact it had on their clinical services.

### Sedentary Behavior and Other Health Outcomes

Overall, participants spent 11 to 12 hours per day sitting (Table 3). The effect size for the reduction in sedentary behavior (minutes per day) was medium (Cohen *d*=0.54) and small for percentage of the day spent in sedentary behavior (Cohen *d*=0.25) at 16 weeks. Using a two-sided significance of *P*<.05 and power of 80%, 110 participants (55 in each group) would be needed to detect a difference in sedentary behavior (minutes per day) of this magnitude between groups, calculated using G*Power version 3.1.9.4. Allowing for a 40% dropout based on this study, 184 participants would need to be recruited (92 in each group) for a randomized controlled trial.

There were statistically significant changes in other health outcomes. There was a significant improvement in functional fitness (6MWT; *P*<.001; Table 4) and quality of life in all domains (MacNew; Table 4). There was also a significant decrease in systolic blood pressure at 6 weeks, which then increased from 6 weeks to 16 weeks (*P*<.05; Table 4).

**Table 3:** Sedentary behavior and physical activity characteristics at baseline, 6 weeks, and 16 weeks.

ActiGraph	Baseline, mean (SD)	6 weeks, mean (SD)	16 weeks, mean (SD)
SB^a^ (minutes per day)	747 (224)	774 (209)	640 (165)
Percentage of SB per day (SB per wear time)	68.2 (9.9)	68.8 (9)	65.7 (9.8)
Duration of SB bouts per day (min)	23 (5.7)	24 (4.8)	22 (4.5)
Number of SB bouts per day	16 (6.5)	17 (7.1)	14 (5.9)
Number of SB breaks per day	15 (6.5)	16 (7.1)	13 (5.9)
MVPA^b^ (minutes per day)	74 (23)	78 (27)	77 (31)
Light physical activity (minutes per day)	257 (78)	261 (78)	253 (84)
VM^c^ (counts per day)	519,365 (127,852)	548,689 (153,920)	535,794 (42,204)
Steps per day	7873 (2073)	8477 (2493)	8028 (2478)
Wear time (minutes per day)	1078 (210)	1113 (208)	970 (179)

^a^SB: sedentary behavior.

^b^MVPA: moderate-to-vigorous physical activity.

^c^VM: vector magnitude.

**Table 4:** Comparison of baseline, 6-week, and 16-week measures.

Outcome	Baseline	6 weeks	16 weeks
Waist circumference (cm), mean (SD)	101 (14)	101 (15)	100 (14)
Waist-to-hip ratio, mean (SD)	0.97 (0.07)	0.96 (0.07)	0.95 (0.08)
BMI (kg/m^2^), mean (SD)	29 (4)	29.1 (4)	28.9 (4.5)
Systolic blood pressure (mm Hg), mean (SD)	119 (12)	113 (11)^a^	118 (13)^b,c^
Diastolic blood pressure (mm Hg), mean (SD)	71 (8)	69 (9)	69 (7)
MacNew^d^ global, mean (SD)	5.3 (0.74)	5.9 (0.75)^e^	5.9 (0.79)^f^
MacNew physical, mean (SD)	5.2 (0.72)	5.9 (0.75)^g^	6 (0.73)^f^
MacNew social, mean (SD)	5.3 (0.97)	6 (0.97)^g^	6.2 (0.92)^f^
MacNew emotional, mean (SD)	5.5 (0.87)	5.9 (0.86)^a^	5.9 (0.94)^b^
HADS^g,h^ anxiety, mean (SD)	4.6 (3.3)	4.3 (4.2)	3.6 (3.3)
HADS depression, median (IQR)	1.5 (1-5.75)	1 (0.25-4.5)	1 (1-5.25)
6-min walk test distance (m), mean (SD)	506 (83)	581 (75)^g^	640 (84)^f,i^

^a^Paired *t* test: baseline to 6 weeks, *P*<.05.

^b^Repeated measures analysis of variance, *P*<.05.

^c^Paired *t* test: 6 weeks to 16 weeks, *P*<.05.

^d^MacNew: MacNew Heart Disease Health-Related Quality of Life Questionnaire.

^e^Paired *t* test: baseline to 6 weeks, *P*<.05.

^f^Repeated measures analysis of variance, *P*<.001.

^g^Paired *t* test: baseline to 6 weeks, *P*<.001.

^h^HADS: Hospital Anxiety and Depression Scale questionnaire.

^i^Paired *t* test: 6 weeks to 16 weeks, *P*<.001.

## Discussion

### Principal Findings

The use of a behavioral smartphone app (Vire) and a web-based behavior change program (ToDo-CR) to decrease sitting time appears feasible in cardiac rehabilitation and may reduce sedentary behavior over time. To our knowledge, this is the first study to report the effects of a behavioral smartphone app and a web-based behavior change program on objectively measured sedentary behavior in cardiac rehabilitation. However, consideration must be given to the number of participants who did not have a smartphone within cardiac rehabilitation. In addition, a smartphone app–based intervention may be more suited to younger cardiac rehabilitation participants. Despite this, even those with a smartphone required support with downloading the app and using the Fitbit, indicating additional support (written materials and telephone support) may be required when implementing a smartphone app–based intervention within this population.

### Comparison With Prior Work

Of the limited studies that have evaluated smartphone app–based interventions in cardiac rehabilitation, descriptions of participant recruitment rates and reasons for study exclusion are limited [21-23]. Varnfield et al [21] reported that 85.6% (715/835) of cardiac rehabilitation patients assessed for the smartphone intervention were ineligible to participate in the study, with not meeting the inclusion criteria cited as the main reason for exclusion (280/715, 39.2%). One of the exclusion criteria was not being able to operate a smartphone for the purposes of the trial; however, the number of patients related to these criteria was not reported. In this study, the number of patients with smartphones was not relevant as all participants were provided with smartphones. More recently, Beatty et al [46] developed a smartphone app to be used in cardiac rehabilitation and reported on its usability. A total of 41 cardiac rehabilitation participants were approached for the app trial and only 2 were excluded because they did not have a smartphone (5%); this was a much lower rate than that found in this study (11/61, 18%).

Regardless of this, the majority of cardiac rehabilitation participants assessed did own a smartphone, as reported in other studies within cardiac rehabilitation settings, and those with a smartphone were significantly younger than participants without a smartphone [18,20,47]. Unsurprisingly, this is a younger cohort compared with previous studies in cardiac rehabilitation [11]. However, this may be the cohort of participants with CHD that need to be targeted with alternative interventions for lifestyle modifications, such as smartphone apps. Despite the steady decline in CHD death rates over the last 40 years in Australia, in more recent years, this decline has slowed in younger age groups (age range 35-64 years), indicating that an increased focus on primary and secondary prevention of heart disease is needed in these age groups [48].

There is some evidence that mobile health (mHealth) technologies, including smartphone apps, can reduce self-reported and objectively measured sedentary behavior levels in healthy populations compared with control groups, although the effect size is small (standardized mean difference −0.26; 95% CI 0.53 to 0.00) [15,49]. The reduction in sedentary behavior effect size reported in the systematic review [49] is of a similar magnitude to that found in this cohort study (Cohen *d*=0.25-0.54). The only study that objectively measured sedentary behavior (ActiGraph accelerometer) in the systematic review found that following a 12-week mHealth intervention for weight reduction, which included a smartphone app, there was a nonsignificant reduction in sedentary behavior in both the intervention and control groups [49]. Despite that specific study being conducted in a different population (university staff and students), there is some indication that mHealth interventions, including smartphone apps, can reduce objectively measured sedentary behavior, suggesting that further investigation of this type of intervention in various populations is warranted. Our study indicates that in cardiac rehabilitation participants, a sample size of 184 participants is necessary for a randomized controlled trial to detect a difference in sedentary behavior (minutes per day) between groups using a smartphone app as the intervention. Furthermore, adequate support (written material and telephone support) is indicated in this population for this type of intervention, which may be as a result of high levels of anxiety and kinesiophobia (fear of movement) in cardiac rehabilitation participants [50,51].

According to these preliminary findings, it is unclear if the 6-week ToDo-CR program was long enough to achieve a sustained change in behavior. According to social cognitive theory, for an increase in physical activity to be adopted and maintained, it must be sustained for at least 6 months [52]. It has been reported that many smartphone apps are not based on behavioral theories and use limited behavior change techniques, particularly for sedentary behavior [13,53]. The ToDo-CR program uses a number of behavior change techniques, including action planning (Do’s), prompting via advice on ways to achieve small actionable goals, opportunities to practice new behaviors, encouraging participants to change their physical and social environments (variety and social opportunity scores), providing feedback on their behavior for self-monitoring over the course of a day or 14 days, and providing rewards with smiley faces if their behavior positively changes from their baseline assessment. By sending behavioral prompts (Do’s), the ToDo-CR program aims to change behavioral habits by disrupting the habits that are common in our daily lives, potentially increasing behavioral or cognitive flexibility, and subsequently changing habits associated with an unhealthy lifestyle [28,32]. It has been suggested that cognitive flexibility is a key mechanism in the reduction of unwanted habits, such as sedentary behavior, and cognitive flexibility can be improved with suitable interventions, resulting in a reduction of habitual sedentary behavior [54]. Thus, this program is based on a behavior change framework and uses behavior change techniques, although a longer program may be necessary to result in changes in sedentary behavior, and further investigation of the potential behavior change mechanism is required.

### Limitations

This study has several strengths, including the use of a personalized smartphone behavior change program based on real-time data analysis and clinical guidelines, the objective measurement of sedentary behavior, and the collection of data to inform a large-scale randomized controlled trial. This study also has several weaknesses. As this was a feasibility study, the sample size was small, and the results should be interpreted with caution. This was also a single-center study where the majority of participants were men, limiting the generalizability of the results within cardiac rehabilitation settings. The ability to detect a significant change in sedentary behavior may have been limited by the small sample size. The attrition rate was high at 16 weeks, although this is commonly reported in app studies targeting the management of disease risk factors and long-term conditions [16]. Further investigation of app engagement using back-end data would have been useful to determine if there was a relationship between app engagement and changes in sedentary behavior. There is some evidence that inexperienced app users may not use all the features of the app and therefore may not receive the proposed benefit from the behavior change smartphone app [55]. In addition, using the last value carried forward for the intention-to-treat analysis may not have been the most appropriate approach to use in this type of research [56]. Finally, as this was a single-cohort study, the detected small to medium effect sizes in reducing sedentary behavior over 16 weeks may not have been related to the ToDo-CR behavior change program and may have resulted from the cardiac rehabilitation program or measurement reactivity [57].

### Conclusions

The behavioral smartphone app (Vire) and web-based behavior change program (ToDo-CR) appear to be feasible and acceptable in cardiac rehabilitation and may be useful to decrease sedentary behavior in this population. Further research is indicated with larger sample sizes, a control group, possibly an extended behavior change program, and longer follow-up to determine whether the behavioral smartphone app and web-based behavior change program decrease sitting time in cardiac rehabilitation participants.

## Appendix

Multimedia Appendix 1 CONSORT-EHEALTH checklist (V 1.6.1).

## Abbreviations

6MWT: 6-min walk test
CHD: coronary heart disease
cpm: counts per minute
HADS: Hospital Anxiety and Depression Scale questionnaire
MacNew: MacNew Heart Disease Health-Related Quality of Life Questionnaire
mHealth: mobile health
MVPA: moderate-to-vigorous physical activity
PCI: percutaneous coronary intervention
UTAUT2: Unified Theory of Acceptance and Use of Technology 2
